# A Case Report of a Cutaneous Mucinous Reaction Associated with Erlotinib Therapy Used in the Treatment of Metastatic Tonsillar Sinus Squamous Cell Carcinoma

**DOI:** 10.4103/JIPO.JIPO_21_19

**Published:** 2019-12-13

**Authors:** Benjamin D Freemyer, Carlos A Torres-Cabala, Anisha B Patel

**Affiliations:** 1 Department of Dermatology, University of Texas MD Anderson Cancer Center, Houston, TX, USA; 2 Department of Pathology, University of Texas MD Anderson Cancer Center, Houston, TX, USA

**Keywords:** Acneiform, adverse drug reaction, cutaneous mucinosis, erlotinib

## Abstract

Cutaneous mucinoses are a diverse group of diseases that are occasionally seen in the context of an adverse drug reaction. We report the case of a 59-year-old male who presented with an asymptomatic, acneiform rash on his forehead, scalp, nose, upper chest, and upper back that had developed after treatment with erlotinib therapy used in the treatment of metastatic tonsillar sinus squamous cell carcinoma. Biopsy of these lesions demonstrated atypical histology that had features of both follicular mucinosis and myxedema. This histologic phenomenon is a rare drug reaction that has not previously been described in association with erlotinib.

## Introduction

The endothelial growth factor receptor (EGFR) is a transmembrane glycoprotein that is found on epithelial cells. These receptors are key signal transducers that can activate multiple intracellular signaling pathways such as the rat sarcoma (RAS)/rapidly accelerated fibrosarcoma, Janus Kinase signal transducer and activator of transcription, and mitogen pathways. These signaling pathways help regulate epidermal proliferation, differentiation, apoptosis, and synthesis of inflammatory cytokines.[1] Overexpression of EGFR has been demonstrated in non-small cell lung cancer, colorectal cancer, squamous cell cancer of the head and neck, breast cancer, and renal cell cancer.[1] One drug class that has been developed to target EGFR is tyrosine kinase inhibitors that inhibit the auto-phosphorylation of EGFR and thus inhibit downstream signaling. Erlotinib is one such drug and was associated with our patient's skin reaction. Erlotinib is now Food and Drug Administration approved for non-small cell lung cancer with EGFR mutations and advanced pancreatic cancer.

Cutaneous toxicities are relatively common side effects among patients receiving EGFR given their key role in epithelial homeostasis. The most common morphologies of cutaneous toxicities reported are acneiform rash, stomatitis, and paronychia.[2] We present a case of acneiform reaction due to erlotinib therapy used in the treatment of metastatic tonsillar sinus squamous cell carcinoma that resolved with a secondary mucinous reaction. This case adds to the range of clinical manifestations presented by cutaneous adverse reactions to EGFR inhibitors. This specific presentation is important as it is clinically profound but has a benign course. It therefore, should not alter management of the underlying malignancy. To our knowledge, this is the first reported case of a cutaneous mucinosis associated with erlotinib treatment.

## Case Report

A 59-year-old Caucasian male had a history of left tonsillar sinus squamous cell carcinoma diagnosed in 2011 with subsequent tonsillectomy and chemoradiation. He had disease recurrence in 2015 with metastasis to the lung, pleura, liver, and bone. A solid tumor genomic assay was performed on the patient's tumor which revealed a BCL2L1 amplification. No EGFR mutations were found. That summer, he initiated erlotinib within a clinical trial and subsequently developed folliculitis on his face, head, chest, and back. His rash improved but did not resolve with doxycycline and topical clindamycin. Erlotinib was discontinued in January 2016 due to disease progression. The rash persisted despite discontinuation of erlotinib and through initiation of nivolumab in February 2016.

He presented to dermatology department in May 2016 due to persistence of his rash. He denied pain, pruritis, and exacerbating factors. He noted stability in distribution of the rash and resolution of a pustular component noted initially. He had no other medical comorbidities. He was taking hydrocodone/paracetamol, ondansetron, and ophthalmic ofloxacin drops. He had no personal or family history of a similar rash. A complete blood count and a complete metabolic panel were within normal limits.

**Figure 1:**
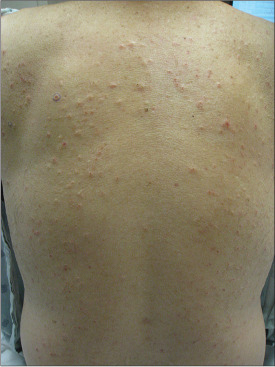
Perifollicular, skin-colored papules both with and without erythematous erosions on the back.

On physical examination, he had perifollicular, erythematous, 1-mm macules on his forehead and anterior scalp. He had one erythematous pustule on his nasal tip. On his upper chest and upper back, he had perifollicular, skin-colored papules with and without erythematous erosions that appeared to be of two distinct morphologies [Figure 1]. The clinical differential diagnosis included folliculitis (however, the pustules had nearly resolved) and acne vulgaris (however, there were no comedones and papules appeared noninflammatory).

**Figure 2:**
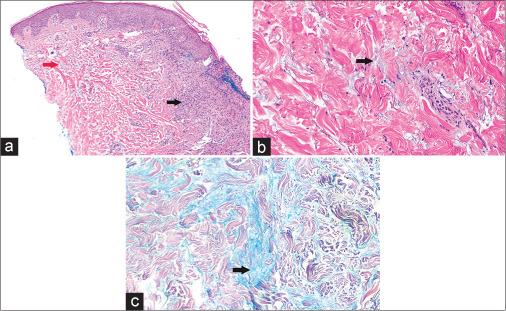
(a) Examination of the skin biopsy reveals granulomatous infiltrate (black arrow) associated with extensive interstitial amphophilic mucin deposition (red arrow) involving superficial, mid, and deep dermis (H and E, ×40). (b) At higher magnification, the presence of amphophilic myxoid material (black arrow) between the thick collagen fibers of the deep dermis, interpreted as mucin, is more evident (H and E, ×200). (c) A colloidal iron special stain highlights abundant bright blue interstitial mucin (black arrow) in deep dermis (colloidal iron, ×200).

Two punch biopsies were obtained: one of an eroded lesion and one of a skin-colored lesion. Clear, mucinous material was secreted upon biopsy of the skin-colored lesion. Histologic findings were consistent between the two biopsies and showed a dermal histiocytic granulomatous infiltrate and abundant mucin between collagen bundles. This was confirmed with a colloidal iron stain [Figure 2]. These histopathologic findings confirmed the diagnosis of cutaneous mucinosis. No further treatment was pursued as the eruption was asymptomatic, and there are no standard treatments for cutaneous mucinosis.

He had a persistent papular eruption on his back at a follow-up with his oncologist in January 2017 despite discontinuing the erlotinib. The patient unfortunately passed away from disease progression in May 2017.

## Discussion

Erlotinib is a reversible and selective inhibitor of EGFR tyrosine kinase that is used to treat a variety of cancers that overexpress EGFR including metastatic squamous cell carcinoma of the head and neck. The most common side effects of erlotinib are diarrhea and cutaneous toxicities. In patients treated with erlotinib, 25%–85% will experience cutaneous toxicities. Most of the toxicities are generally mild with Common Terminology Criteria for Adverse Events Grade 3 toxicities occurring in 1%–10% of patients.[2] The median time to the onset of cutaneous toxicity varies by which morphology is observed. The median time to the development of rash, pruritus, and paronychia is 8, 11, and 32 days, respectively.[3] To our knowledge, a cutaneous toxicity to erlotinib with a mucinous morphology has not previously been described.

Cutaneous mucinoses are a diverse group of diseases, in which mucin accumulates within the skin or the hair follicle in a diffuse or focal pattern. They are classified as either primary, where mucin deposition is the primary histologic feature, or secondary, where mucin deposition is just an associated histologic finding. The cause of excess mucin deposition in the skin is not entirely known, but circulating cytokines such as interleukin-1, tumor necrosis factor-alpha, and transforming growth factor-beta may play a role. Cutaneous mucin deposition is rare as an adverse drug reaction, but has been reported.[4,5]

Secondary follicular mucinoses are a group of disorders where mucin deposition within hair follicles presents as a histologic phenomenon in reaction to a separate primary process. Secondary follicular mucinous reactions have been reported in association with infliximab and leuprolide acetate.[4,5] In these reactions, it was postulated that a perturbation of lymphocyte function or an immune privilege collapse within the hair follicles played a role in the pathogenesis. Secondary dermal mucinous reactions have also been observed in patients receiving subcutaneous interferon injections.[6] The dermal mucin deposition observed in these cases was thought to be explained by the capability of interferon beta-1b and interferon-alfa to stimulate fibroblasts, increasing mucin deposition.

Underlying follicular mucin deposition has been previously reported in patients who presented clinically with an acneiform rash.[7–10] In none of these cases was a drug implicated as the cause and in only one of the cases was the patient noted to have a history of acne. We were unable to find a case where acne vulgaris transformed to or resolved with follicular mucin deposition.

There is no standard treatment for cutaneous mucin deposition as an adverse drug reaction. In many cases, simply discontinuing the drug responsible for the rash has been shown to lead to resolution.[8] One case also demonstrated that if the insulting drug is continued, the rash is unlikely to resolve even if it is simultaneously treated with topical corticosteroids.[5] Our case demonstrates that the natural history of erlotinib-induced cutaneous mucinosis is persistence, even after erlotinib is discontinued. This is in contrast to other reported cases where withdrawal of the inciting medication leads to resolution of the cutaneous mucinous reaction.[4,6]

Erlotinib is known to cause various cutaneous toxicities. Our case is noteworthy because it demonstrates a unique morphology of cutaneous toxicity that has not previously been associated with erlotinib.
